# Associations Between Mental Health Problems in Adolescence and Educational Attainment in Early Adulthood: Results of the German Longitudinal BELLA Study

**DOI:** 10.3389/fped.2022.828085

**Published:** 2022-02-25

**Authors:** Carina Meißner, Ann-Katrin Meyrose, Anne Kaman, Martha Michalkiewicz, Ulrike Ravens-Sieberer

**Affiliations:** ^1^Department of Child and Adolescent Psychiatry, Psychotherapy, and Psychosomatics, University Medical Center Hamburg-Eppendorf, Hamburg, Germany; ^2^Clinical Psychology, Helmut-Schmidt-University/University of the Federal Armed Forces Hamburg, Hamburg, Germany; ^3^Institute for Experimental Psychology, Heinrich Heine University Düsseldorf, Düsseldorf, Germany

**Keywords:** externalizing problems, internalizing problems, youth, young adults, educational attainment, longitudinal study

## Abstract

Mental health problems (MHP) in adolescence are a major public health concern of the 21st century. Global prevalence estimates range between 10 and 20%. Most MHP manifest by adolescence and persistence rates are high, often accumulating further impairment in early adulthood and beyond. We analyzed data of *N* = 433 participants from the German longitudinal BELLA study to examine whether MHP in adolescence negatively affect educational attainment in early adulthood. Externalizing and internalizing MHP among adolescents aged 11–17 years were assessed at baseline using the Strengths and Difficulties Questionnaire. Educational attainment was assessed at the 6-year follow-up based on level of education, failure to attain the expected level of education, and dropout from vocational or academic training. Findings from logistic regression analyses suggest that more pronounced externalizing MHP in adolescence predict a lower level of education in early adulthood. We did not find a corresponding effect for internalizing MHP. Adolescents with higher-educated parents were less likely to attain a lower level of education themselves and less likely to fail in attaining their expected level of education. Our findings support that educational attainment presents a central channel for intergenerational reproduction of education and forms an important pathway for upward, but also downward social mobility. The current study emphasizes school as a central setting to implement measures to prevent onset and persistence of MHP and to foster equal opportunities in education.

## Introduction

Mental health problems (MHP) among adolescents represent a core health challenge of the 21st century (1, 2). Estimates of global prevalence are high and range between 10 and 20% (3, 4). MHP can cause persistent impairment for the individual (5) and create high societal and economic burden for health, welfare, and educational systems (5–7). Dichotomizing MHP into externalizing vs. internalizing MHP allows a more precise understanding of possible implications (8, 9). While externalizing MHP encompass hyperactivity and conduct problems typically directed toward others, internalizing MHP comprise inwards-oriented symptoms of anxiety, depression, and social difficulties (10, 11). In Germany, the estimated prevalence of MHP among children and adolescents is 17.6% (12). Prevalence estimates of externalizing MHP range from 2.0 to 5.7% for hyperactivity problems and 12.2% for conduct problems, while prevalence estimates of internalizing MHP range from 11.2 to 16.1% for depression and 10.6 to 15.1% for anxiety among children and adolescents between 7 and 19 years (13). Most externalizing and internalizing MHP manifest during adolescence – 50.0% by age 14 and 79.5% by age 17—with a high tendency to persist into adulthood (5, 14).

The transition from adolescence to early adulthood is a vulnerable phase for the manifestation and chronification of MHP, further aggravated by multiple developmental challenges (15, 16). It coincides with the major educational transition from secondary school education to vocational or academic training, while facing increasing autonomy and identity formation (17), and developing occupational aspirations (18). Educational attainment during this transition determines later opportunities in life (19, 20). The higher the school-leaving certificate attained in adolescence, the wider the range of vocational options in early adulthood, and the more likely favorable socioeconomic outcomes and improved social mobility become (20–22). Shortfalls in educational attainment are costly and governments invest high expenses to compensate for resulting deficits (23). Higher educational attainment enhances health literacy and behavior as well as physical and mental health (23, 24). Such enhancement effects were found to transfer across generations (25–27). Understanding educational attainment as an essential resource to individual and societal well-being underlines the importance of adolescents thriving in their transition to early adulthood (16, 28).

Reviews indicate that adolescents with externalizing and internalizing MHP are at risk for attaining poorer educational outcomes (29–31). While externalizing and internalizing MHP express themselves differently in terms of visibility (32, 33), symptoms can overlap and it proves insightful to assess their relative influence on later educational outcomes (34). When assessed simultaneously, both externalizing and internalizing MHP among adolescents were associated with impaired school and academic performance, i.e., lower math and reading achievements among U.S. students (35, 36), a lower likelihood of graduating from U.S. high schools (37), and lower academic functioning assessed by college aptitude scores among Belgian University students (38). Melkevik et al. (39) report in their review that internalizing MHP were repeatedly found to be associated with an increased risk of dropout from education, but that this association was partly accounted for by co-occurring externalizing MHP. According to a meta-analysis by Riglin et al. (40), internalizing MHP were associated with lower school grades and higher rates of dropout, but associations were less consistent for anxiety than for depression. Breslau et al. (41) found that only externalizing MHP remained significantly associated with dropout from or interruption of U.S. high school education after adjusting for internalizing MHP. To date, research reports stronger and more consistent effects of externalizing MHP on educational attainment compared to a more mixed picture for internalizing MHP (30, 34).

While reviews by Hale et al. (29) and Dadaczynski (30) conclude that much of the research to date has been conducted in the United States and, accordingly, in the U.S. educational system, findings from European studies confirm that externalizing and internalizing MHP may put adolescents at risk of attaining poorer educational outcomes. Results from a Dutch prospective cohort study suggest that a low level of education at age 19 is predicted by externalizing MHP at age 11, an increase of externalizing MHP between ages 11 and 16, and among female adolescents also by an increase of internalizing MHP (42). Likewise, Patalay et al. (43) found that increasing trajectories of internalizing MHP between ages 11 and 14 negatively affected academic attainment in national standardized tests in England. Jonsson et al. (44) reported that depression among Swedish adolescents was associated with a reduced likelihood of graduating from higher education, and that this link in males was not fully accounted for by early school performance, maternal education, and socioeconomic status (SES). Evensen et al. (8) found that, in a Norwegian population-based sample, externalizing MHP among adolescents predicted a reduced number of completed years of education, increased the likelihood of school dropout, and lowered chances of enrolling for academic training – despite stable family-level characteristics shared by siblings. On the contrary, internalizing MHP were associated with lower educational attainment when assessed alone, but positively related when controlling for co-morbidity with externalizing MHP. In contrast, Mikkonen et al. (34) showed that, in a register-based sample of Finnish adolescents, internalizing MHP were negatively associated with educational attainment regardless of co-occurring externalizing MHP. Further longitudinal research on the detrimental impact of externalizing and internalizing MHP on educational attainment is needed and contributing factors should be considered (29–31).

MHP and educational attainment are affected by age, gender, migration background, and SES. First, trajectories of externalizing and internalizing MHP vary with age and gender (45–47). While externalizing MHP are more frequent among males and decrease during adolescence, internalizing MHP are more frequent among females and increase during adolescence (46). In addition, gender roles affect ratings and opportunities in education (21, 23). Second, having a migration background is associated with both a higher risk of MHP and disadvantaged opportunities in education (48–50). Third, the prevalence of MHP in adolescence follows a social gradient: the lower the SES as indicated by the level of parental education, household income, and parental status of employment, the higher the risk of being affected by MHP (27, 51). Indicators of SES—including level of parental education—predict educational attainment, which in turn determines later socioeconomic well-being (52). Educational attainment therefore forms the main pathway for up- or downward social mobility across generations (52–54).

Against this background, we examined the association between MHP and educational attainment considering all above-mentioned factors. To our knowledge, this is the first study to investigate the impact of MHP on educational attainment during the transition from adolescence to early adulthood in Germany. In the present study, we assessed externalizing and internalizing MHP among adolescents simultaneously and examined their effects on the attained level of education, failure to attain the expected level of education, and dropout from vocational or academic training using longitudinal population-based data. First, we hypothesized that more pronounced externalizing and internalizing MHP in adolescence increase the likelihood of attaining a lower level of education in early adulthood. Second, we expected that more pronounced externalizing and internalizing MHP increase the likelihood of failing to attain the level of education that the participant expected. Third, we assumed that more pronounced externalizing and internalizing MHP increase the likelihood of dropout from vocational or academic training. We included age, gender, the interaction of age and gender, migration background, and three indicators of SES (i.e., level of parental education, household income, and parental status of employment) as control variables in all analyses.

## Materials and Methods

### Design and Procedure

Data were derived from the population-based BELLA cohort study in Germany. BELLA represents the mental health module within the German National Health Interview and Examination Survey for Children and Adolescents (KiGGS). The Robert Koch Institute (RKI, Federal Public Health Institute of Germany) conducts the KiGGS study with the main objective to obtain central indicators of physical and mental health, health behaviors, and health service utilization among children and adolescents in Germany (55–57). The BELLA study gathers complementary data on mental health and well-being and is carried out by the University Medical Center Hamburg-Eppendorf (58–61).

Using a multistage random sampling procedure, KiGGS comprised a representative sample of *n* = 17,641 children and adolescents aged 0 to 17 years from 167 sampling points across Germany at baseline. The KiGGS baseline assessment was conducted between 2003 and 2006. For the BELLA baseline study (2003–2006), a random subsample of KiGGS participants was drawn, including *n* = 2,863 children and adolescents aged 7–17 years. Both KiGGS and BELLA are designed as prospective longitudinal studies with several follow-up assessments, including a 6-year follow-up between 2009 and 2012. Analyses in the present study use data from the baseline assessment (also referred to as T0) and the 6-year follow-up (also referred to as T1) of the BELLA study.

Data were collected through standardized computer-assisted telephone interviews (CATI); subsequent questionnaires were provided to the parents and participating adolescents if aged 11 years or older. In accordance with the Declaration of Helsinki, parents provided written informed consent for all participants, while participants aged 14 years or older gave additional written informed consent. Approval was granted by the ethics committee of the University Hospital Charité Berlin and by the Federal Commissioner for Data Protection in Germany. A thorough description of the KiGGS study is published elsewhere (55–57). For further details regarding design and methods of the BELLA cohort study, see Ravens-Sieberer et al. (58–60) and Otto et al. (61).

### Sample

**Figure 1:**
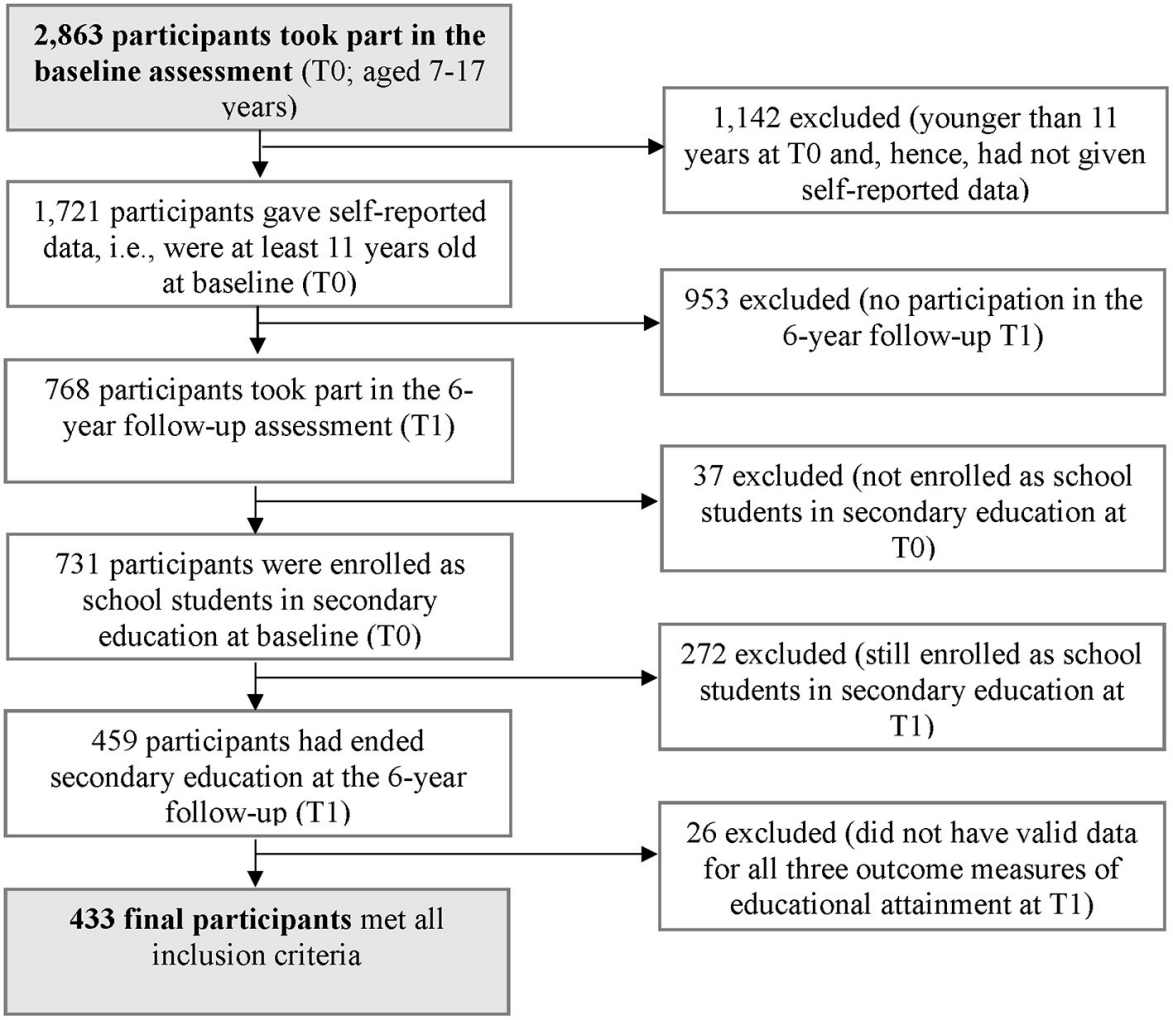
Flow chart of the selection procedure for study participants.

Of the 2,863 children and adolescents who participated in the BELLA baseline assessment, 433 participants met inclusion criteria for the present study (see Figure 1). Adolescents were included if they (i) were at least 11 years old and had given self-reported data at baseline T0 (included: *n* = 1,721), (ii) had participated in the 6-year follow-up T1 between the ages of 18–23 years old, (included: *n* = 768), (iii) were school students at T0 (German *Gymnasium, Realschule* oder *Hauptschule*; included: *n* = 731), and (iv) were no longer enrolled as school students 6 years later at T1 (included: *n* = 459). Finally, participants were included if they (v) had valid data for all three outcome measures of educational attainment (included: *n* = 433). We could finally analyze data from 433 participants.

### Instrument

#### Externalizing and Internalizing MHP

*Externalizing and internalizing MHP* were operationalized based on the self-reported Strengths and Difficulties Questionnaire (SDQ-S; German version) (62, 63). The SDQ-S is a well-established, reliable screening questionnaire for assessing MHP among adolescents aged 11–17 years (45). It consists of 25 items answered on a three-point response scale (0 = *not true*; 1 = *somewhat true*; 2 = *certainly true*) referring to the previous 6 months. In the present study, the *Emotional Symptoms* and *Peer Problems* subscales were added to yield the *internalizing MHP score*. Likewise, subscales on *Hyperactivity* and *Conduct Problems* yielded the *externalizing MHP score* (10, 64). Externalizing and internalizing MHP scores range from 0 to 20 points each, with higher scores indicating more pronounced MHP. Previous research demonstrated satisfactory internal consistency for the externalizing MHP score (α = 0.76; 10) and close to satisfactory for the Internalizing MHP score (α = 0.66; 10). In this study, internal consistency was close to satisfactory for both scores (α = 0.65 and α = 0.69, respectively).

#### Lower Level of Education

The level of education at the 6-year follow-up was based on the attained school-leaving certificate and dichotomized into a *lower level of education* (reference category) vs. a *higher level of education*. Operationalization followed the CASMIN scheme for educational classification (65–67). The lowest German school-leaving certificate is the secondary general school-leaving certificate, i.e., *Hauptschulabschluss*, which covers 9 years of compulsory schooling and provides access to basic vocational training under the dual system. The intermediate German school-leaving certificate, i.e., *Realschulabschluss*, covers 10 years of extended general education and grants access to a much wider range of vocational training programs and options (vocational training under the dual system, full-time school-based vocational training, or further schooling and subsequent academic training in lower tertiary education). The highest German school-leaving certificate is the higher education entrance qualification, i.e., *Abitur*, which is awarded after completing a 13-year track of deepening general education. It prepares students to enter academic training in higher tertiary education and entitles holders to the widest range of vocational options (19, 65, 68). Participants who had attained the lowest school-leaving certificate or reported dropout from school were gathered into the group with a *lower level of education*. Participants who had attained intermediate and highest school-leaving certificates were gathered into the group with a *higher level of education*. Specific information on school-leaving certificates from other types of schools such as comprehensive or special-needs schools was not available.

#### Failure to Attain the Expected Level of Education

Participants' failure to attain their expected level of education was assessed at the 6-year follow-up based on the self-developed item “I (at least) attained the school-leaving certificate that I had intended to achieve.” Participants indicated responses on a three-point response scale as either 1 = *yes, fully applies*, 2 = *yes, partially applies*, or 3 = *no, does not apply*. For the present analysis, responses were dichotomized [*no, does not apply* and *yes, partially applies* were coded as (*partial*) *failure*; *yes, fully applies* was coded as *no failure*]. *Failure to attain the expected level of education* was operationalized as *yes* (reference category) vs. *no*.

#### Dropout From Vocational or Academic Training

Dropout from vocational or academic training was assessed at the 6-year follow-up based on the self-developed item “*I have terminated a course of education before its regular completion*.” In Germany, a course of education generally refers to basic or extensive vocational training, or academic training in tertiary education. Possible responses on a three-point response scale were 1 = *yes, fully applies*, 2 = *yes, partially applies*, or 3 = *no, does not apply*. Responses were dichotomized [*yes, fully applies* and *yes, partially applies* were coded as (*partial*) *dropout; no, does not apply* was coded as *no dropout*]. *Dropout from vocational or academic training* was operationalized as *yes* (reference category) vs. *no*.

#### Sociodemographic Variables

Age (in exact years), gender [1 = *male* (reference category), 0 = *female*], the interaction term age by gender, migration background, and three indicators of SES were all assessed at baseline. *Migration background* was assessed based on Schenk et al. (50). Migration background was determined as *migrant* (i.e., participants with a two-sided migration background; reference category) or *non-migrant* (i.e., participants with no or a one-sided migration background). The three separate, parent-reported indicators of SES were: level of parental education, household income, and parental status of employment. *Level of parental education* was operationalized as the mean of maternal and paternal years of education, following Reiss et al. (27). To estimate completed years of education, the standard number of years to attain a certain German school-leaving certificate in secondary education and the standard number of years to acquire a certain vocational qualification were added, ranging up to 18 possible years in total. *Household income* was measured based on the family's approximate monthly net equivalent income (in €) and adjusted for the size of the household and the age of its members based on a modified equivalence scale by the Organization for Economic Co-Operation and Development (OECD modified equivalence scale: head of household = 1, each additional adult household member = 0.5, children = 0.3) (69, 70). *Parental status of employment* was operationalized as either *at least one parent unemployed* (reference category) or *both parents employed* (27).

### Statistical Analysis

We described our sample under analysis using frequencies, means and standard deviations of relevant variables. We compared our study sample (i.e., *n* = 433) to BELLA baseline participants with the same age range, but not meeting the inclusion criteria (i.e., *n* = 1,288). To investigate bivariate associations, we calculated Pearson's correlation coefficients. To evaluate the strength of bivariate associations, we followed Cohen (71): a correlation of *r* = 0.10 indicates a small, *r* = 0.30 a moderate, and *r* = 0.50 a strong relationship.

#### Logistic Regression Analysis

For our main analyses, we ran three binary multiple logistic regression models to test our three hypotheses covering the impact of externalizing and internalizing MHP on different measures of educational attainment. Model A served to predict the likelihood of attaining a lower level of education. Model B was used to investigate effects on failing to attain the expected level of education. Model C examined effects on dropout from vocational or academic training. All analyses followed a hierarchical approach. First, all control variables, i.e., age, gender, the interaction term age by gender, migration background, and the three indicators of SES (level of parental education, household income, and parental status of employment) were entered. Second, externalizing and internalizing MHP were entered simultaneously into the model. Following this proceeding, we were able to assess the improvement in model fit due to externalizing and internalizing MHP. Final models on lower level of education (Model A), failure to attain the expected level of education (Model B), and dropout (Model C) are reported.

To determine goodness of fit, the likelihood ratio was interpreted. Improvement in model fit due to adding externalizing and internalizing MHP as predictors was assessed based on the likelihood ratio (final model—model with constant and control variables included), as indicated by the χ^2^-statistic. Additionally, Nagelkerke's *R*^2^ is reported for the final model and ΔNagelkerke's *R*^2^ (final model—model with constant and control variables included) for the according increase in Nagelkerke's *R*^2^ by adding externalizing and internalizing MHP as predictors. Following Muijs (72), a Nagelkerke's *R*^2^ of <0.10 indicated *poor* improvement in model fit over the baseline model, a value between 0.10 and 0.30 *modest*, a value between 0.30 and 0.50 *moderate*, and a value >0.50 *strong* improvement.

To assess the variables of our interest, regression coefficients, standard errors, and *p*-values were interpreted and odds ratios (*OR*) and the corresponding 95% confidence intervals (*CI*) were examined. Before analysis, all metric variables (i.e., age, level of parental education, household income, externalizing, and internalizing MHP) were centered on the sample grand mean. Missing values were below 2% for control variables and predictors and could be replaced using the Expectation-Maximization (EM) algorithm.

In order to test robustness of results, sensitivity analyses were conducted without imputation of missing data. Concerning attainment of a lower level of education (A), we reiterated our analysis without participants aged 16–17 years at baseline assuming they might already be on the higher educational track. Furthermore, analyses of failure to attain the expected level of education (B) and of dropout (C) were reiterated with different coding schemes (i.e., with *yes, partially applies*-responses excluded and with *yes, partially applies* responses coded as the opposite to the original coding scheme, respectively). Finally, assumptions of logistic regression analysis were examined. All analyses were conducted using IBM SPSS Statistics Version 22 and the significance level was determined as *p* < 0.05 for all analyses.

#### Additional Analysis

We conducted the following additional analyses: In order to allow for a more precise investigation of the effects of externalizing and internalizing MHP on attaining a lower level of education (A), we modified and reiterated the hierarchical analysis by entering externalizing and internalizing MHP separately instead of simultaneously. We reiterated hierarchical analyses concerning all three models on attaining a lower level of education (A), failure to attain the expected level of education (B), and dropout (C) with the interaction terms externalizing MHP by gender, internalizing MHP by gender, externalizing MHP by age, and internalizing MHP by age entered simultaneously as predictors in an additional, final step. Regarding dropout from vocational or academic training (C), we conducted an additional analysis to examine participants' subjective causal attributions about the relationship between MHP and reported dropout. Specifically, data from three items, all assessed at the 6-year follow-up, was used: (i) “*I have or have had MHP that caused me to fall short of my potential in a course of education*,” (ii) “*I have or have had MHP that threaten or have threatened successful completion of a course of education*,” and (iii) “*I have or have had MHP that led me to drop out from a course of education before its regular completion*.” Possible responses on a three-point response scale were 1 = *yes, fully applies*, 2 = *yes, partially applies*, or 3 = *no, does not apply* and were dichotomized as (*partially*) *yes* vs. *no*. Frequencies, Chi-squared tests and Fisher's exact test were computed to compare groups that did vs. did not report dropout.

## Results

### Sample Characteristics

Characteristics of the investigated sample at baseline and the distributions of the three outcome measures at the 6-year follow-up assessment are presented in Table 1. The sample consisted of 433 participants, of which 237 (54.7%) were female. Age at baseline ranged from 11.01 to 17.98 years (*M* = 14.88, *SD* = 1.79). Of all participants, 6.7% had a two-sided migration background, slightly below the percentage of 8.8% in the German population in 2003 (73). Concerning SES at baseline, parental years of education were 13.17 years (*SD* = 2.33) on average, which roughly corresponds to the German average of 12.65 years in 2000 (74). Mean equivalent household net income was 1,241€ per month (*SD* = 555), falling below the German average of 1,875€ per month in 2003 (73). Moreover, 11.3% of all participants lived in families with at least one unemployed parent at baseline, corresponding to the German rate of unemployment of 11.7% in 2005 (74). Regarding externalizing and internalizing MHP, mean scores at baseline were 5.09 (*SD* = 2.70) and 4.27 (*SD* = 2.85), respectively. By the 6-year follow-up, 6.9% of all participants had attained a lower level of education, 14.8% reported failure to attain their expected level of education, and 12.5% reported dropout from vocational or academic training before its regular completion.

**Table 1:** Sample characteristics at baseline (T0) and outcome measures at 6-year follow-up (T1).

	**Adolescents (*****N*** **=** **433) at baseline (T0)**				
	***n***	**%**	***M* (*SD*)**	**Potential range**	**Observed range**
Gender	433				
*Male*	196	45.3			
*Female*	237	54.7			
Age (in completed years with two decimals)	433		14.88 (1.79)	11.00–17.99	11.01–17.98
Migration background	433				
*Yes*	29	6.7			
*No*	404	93.3			
Parental education (in years)	427		13.17 (2.33)	8.00–18.00	8.50–18.00
Household income (in 100€)	427		12.41 (5.55)	>0.00	3.02–37.42
Parental status of employment	432				
*At least one parent unemployed*	49	11.3			
*None unemployed*	383	88.7			
Externalizing MHP score	432		5.09 (2.70)	0–20	0–14
*Hyperactivity Scale*	432		3.31 (1.94)	0–10	0–9
*Conduct Problems Scale*	432		1.78 (1.34)	0–10	0–7
Internalizing MHP score	432		4.27 (2.85)	0–20	0–18
*Emotional Problems Scale*	432		2.34 (1.98)	0–10	0–9
*Peer Problems Scale*	432		1.93 (1.55)	0–10	0–10
	**Early adults (*****N*** **=** **433) at the 6-year follow-up (T1)**				
	***n***	**%**	***M*** **(*****SD*****)**	**Potential range**	**Observed range**
Level of education	430				
*Lower*	30	6.9			
*Higher*	403	93.1			
Failure to attain the expected level of education	433				
*Yes*	64	14.8			
*No*	369	85.2			
Dropout from vocational/academic training	433				
*Yes*	54	12.5			
*No*	379	87.5			

*Missing values were given for n = 6 on parental education, n = 6 on household income, n = 1 on parental status of employment, n = 1 on the Externalizing MHP score, and n = 1 on the Internalizing MHP score. Responses regarding attainment of the expected level of education were n = 369 for yes, fully applies, n = 53 for yes, partially applies, and n = 11 for no, does not apply. Responses regarding dropout from vocational or academic training were n = 29 for yes, fully applies, n = 25 for yes, partially applies, and n = 379 for no, does not apply. Responses were dichotomized. For more information, see Section Instrument*.

*MHP = mental health problems*.

We found small differences between our study sample under analysis and BELLA baseline participants with the same age range, but not meeting inclusion criteria [results presented in Supplementary Table 1; *p* was Holm-Bonferroni corrected (75, 76)]. In comparison, our study sample was older and included more females. Additionally, our sample had a slightly higher level of parental education and household income, and lower rate of parental unemployment, and included less participants with migration background. Our sample reported a little less pronounced self-reported externalizing MHP.

### Bivariate Associations

A correlation matrix of bivariate associations between externalizing MHP, internalizing MHP, all control variables, and the outcome measures is presented in the Supplementary Table 2. Externalizing MHP were positively associated with attaining a lower level of education and with failure to attain the expected level of education. Effect sizes were small and significant (*r* = 0.19, *p* < 0.001 and *r* = 0.14, *p* = 0.003, respectively). Externalizing MHP and dropout from vocational or academic training were not significantly associated (*r* = −0.05, *p* = 0.340). Internalizing MHP were neither significantly associated with attaining a lower level of education (*r* = −0.01, *p* = 0.912), nor with failure to attain the expected level of education (*r* = 0.09, *p* = 0.062), nor with dropout from vocational or academic training (*r* = 0.09, *p* = 0.054).

### Logistic Regression Analyses

We ran thee binary logistic regression analyses to test whether more pronounced externalizing and internalizing MHP in adolescence increased the likelihood of (i) attaining a lower level of education in young adulthood (model A), (ii) failing to attain the expected level of education (model B), and (iii) dropout from vocational or academic training before its regular completion (model C). Results of the final models are reported in Table 2.

**Table 2:** Final models predicting lower level of education **(A)**, failure to attain the expected level of education **(B)**, and dropout from vocational or academic training **(C)**.

	***B***	***SE***	***p***	***OR***	**95% CI of *OR***
**Model A: lower level of education**					
Externalizing MHP (centered)	0.23	0.08	**0.003**	**1.26**	[1.08, 1.46]
Internalizing MHP (centered)	−0.05	0.08	0.576	0.95	[0.81, 1.12]
Age (in years; centered)	−0.34	0.18	0.052	0.71	[0.50, 1.00]
Gender (male)	0.58	0.48	0.226	1.79	[0.70, 4.60]
Age*Gender	0.19	0.24	0.433	1.21	[0.76, 1.93]
Migration background (yes)	1.08	0.67	0.105	2.94	[0.80, 10.82]
Parental education (in years; centered)	−0.71	0.20	**<0.001**	**0.49**	[0.33, 0.72]
Household income (in 100€; centered)	−0.08	0.07	0.270	0.93	[0.81, 1.06]
Parental status of employment (at least one parent unemployed)	0.57	0.58	0.327	1.76	[0.57, 5.48]
χ^2^ (9, *N* = 433) = 57.57, *p*<**0.001**, Nagelkerke's *R*^2^ = 0.32					
**Model B: failure to attain the expected level of education**					
Externalizing MHP (centered)	0.10	0.05	0.056	1.11	[1.00, 1.22]
Internalizing MHP (centered)	0.08	0.05	0.109	1.08	[0.98, 1.19]
Age (in years; centered)	−0.26	0.11	**0.017**	**0.77**	[0.63, 0.96]
Gender (male)	0.05	0.31	0.877	1.05	[0.58, 1.91]
Age*Gender	0.13	0.16	0.401	1.14	[0.84, 1.56]
Migration background (yes)	0.26	0.55	0.632	1.30	[0.45, 3.80]
Parental education (in years; centered)	−0.22	0.09	**0.010**	**0.80**	[0.68, 0.95]
Household income (in 100€; centered)	0.01	0.03	0.881	1.01	[0.94, 1.08]
Parental status of employment (at least one parent unemployed)	−0.22	0.47	0.635	0.80	[0.32, 2.01]
χ^2^ (9, *N* = 433) = 29.50, *p* = **0.001**, Nagelkerke's *R*^2^ = 0.12					
**Model C: dropout from vocational or academic training**					
Externalizing MHP (centered)	−0.08	0.06	0.181	0.92	[0.82, 1.04]
Internalizing MHP (centered)	0.11	0.05	**0.029**	**1.12**	[1.01, 1.24]
Age (in years; centered)	0.11	0.12	0.363	1.12	[0.88, 1.41]
Gender (male)	0.20	0.32	0.526	1.23	[0.66, 2.29]
Age*Gender	0.22	0.18	0.219	1.24	[0.88, 1.76]
Migration background (yes)	−0.95	0.78	0.224	0.39	[0.09, 1.78]
Parental education (in years; centered)	−0.02	0.08	0.828	0.98	[0.84, 1.15]
Household income (in 100€; centered)	−0.03	0.04	0.388	0.97	[0.90, 1.04]
Parental status of employment (at least one parent unemployed)	0.41	0.44	0.347	1.51	[0.64, 3.55]
*χ^2^ (9, N = 433) = 16.82, p = 0.052, Nagelkerke's R*^2^ *= 0.07*.					

*MHP = mental health problems; significant p-values and corresponding Odds Ratios (OR) in bold*.

#### Lower Level of Education

Externalizing and internalizing MHP and all control variables explained Nagelkerke's *R*^2^ = 0.32 in model A on predicting a lower level of education. This indicates a moderate improvement in model fit over the baseline model with only the constant included; χ^2^ (9, *N* = 433) = 57.57, *p* < 0.001. Externalizing MHP significantly predicted the likelihood of attaining a lower level of education, *b* = 0.23, Wald χ^2^ (1, *N* = 433) = 8.88, *p* = 0.003. With each additional point of the adolescent's externalizing MHP score, the odds of attaining a lower level of education increased by 1.26. Therefore, with more pronounced externalizing MHP, the likelihood of attaining a lower level of education increases. No significant effect was found for internalizing MHP, *b* = −0.05, Wald χ^2^ (1, *N* = 433) = 0.31, *p* = 0.576. Level of parental education significantly predicted the likelihood of attaining a lower level of education, *b* = −0.71, Wald χ^2^ (1, *N* = 433) = 12.90, *p* < 0.001. With each additional year of parental education, the change in odds of attaining a lower level of education was 0.49 and, thus, with increasing level of parental education, adolescents themselves were less likely to attain a lower level of education.

In order to examine effects of externalizing and internalizing MHP, the predictors of our main interest, on attaining a lower level of education more closely, we compared final model A with all variables entered to the intermediate model with only the control variables and constant entered. Adding externalizing and internalizing MHP as predictors yielded a significantly improved model fit of the final model compared to the intermediate model with only the constant and control variables included, χ^2^ (2, *N* = 433) = 9.30, *p* = 0.010, with a ΔNagelkerke's *R*^2^ of.05 (not presented in Table 2).

##### Sensitivity and Additional Analysis

We reran Model A with participants aged 16–17 years at baseline excluded, confirming our results [externalizing MHP: *b* = 0.25, Wald χ^2^ (1, *N* = 306) = 7.17, *p* = 0.007, *OR* = 1.28; level of parental education: *b* = −0.90, Wald χ^2^ (1, *N* = 306) = 11.67, *p* = 0.001, *OR* = 0.41] and rendering the effect of age significant [*b* = −0.72, Wald χ^2^ (1, *N* = 306) = 6.18, *p* = 0.013, *OR* = 0.49; further results not reported].

As we were interested in a more precise investigation of the separate effect sizes of externalizing and internalizing MHP on the level of education, we reran Model A with control variables entered to the model first. However, in the second step only internalizing MHP were entered. In a third and final step, externalizing MHP were entered to the model. Results revealed a significant and relevant improvement in model fit after entering externalizing MHP as a predictor, χ^2^ (1, *N* = 433) = 9.29, *p* = 0.002, with a ΔNagelkerke's *R*^2^ of 0.05 (not presented in Table 2).

In addition, we reiterated Model A with the interaction terms externalizing MHP by gender, internalizing MHP by gender, externalizing MHP by age, and internalizing MHP by age entered simultaneously as predictors in a final step. This modification rendered the effect of externalizing MHP insignificant (*p* = 0.108), but yielded a significant effect of the interaction of internalizing MHP by age on attaining a lower level of education [*b* = −0.13, Wald χ^2^ (1, *N* = 433) = 4.19, *p* = 0.041]. As before, level of parental education significantly predicted the likelihood of attaining a lower level of education [*b* = −0.70, Wald χ^2^ (1, *N* = 433) = 11.97, *p* < 0.001]. In addition, migration background significantly predicted the likelihood of attaining a lower level of education [*b* = 1.53, Wald χ^2^ (1, *N* = 433) = 4.62, *p* =0.032; results presented in Supplementary Table 3].

#### Failure to Attain the Expected Level of Education

In model B on predicting the likelihood of failure to attain the expected level of education, externalizing and internalizing MHP and all control variables explained Nagelkerke's *R*^2^ = 0.12. This indicates a modest improvement in model fit over the baseline model with only the constant included; χ^2^ (9, *N* = 433) = 29.50, *p* = 0.001. Neither externalizing MHP nor internalizing MHP significantly predicted the likelihood of not attaining the expected level of education, *b* = 0.10, Wald χ^2^ (1, *N* = 433) = 3.65, *p* = 0.056 and *b* = 0.08, Wald χ^2^ (1, *N* = 433) = 2.57, *p* = 0.109, respectively. Level of parental education significantly predicted the likelihood of not attaining the expected level of education, *b* = −0.22, Wald χ^2^ (1, *N* = 433) = 6.63, *p* = 0.010. With each additional year of parental education, the change in odds of not attaining the expected level of education was 0.80 and, thus, with increasing level of parental education, adolescents themselves were less likely to fail in attaining their expected level of education.

To examine the effects of externalizing and internalizing MHP, the predictors of our main interest, on the expected level of education more closely, we compared final model B with all variables entered to the intermediate model with only the control variables and constant entered. Adding externalizing and internalizing MHP as predictors yielded a significantly improved model fit of the final model compared to the intermediate model with only the constant and control variables included, χ^2^ (2, *N* = 433) = 7.78, *p* = 0.020, with a ΔNagelkerke's *R*^2^ of .03 (not presented in Table 2).

##### Sensitivity and Additional Analysis

To test robustness of results, we reran model B with different coding schemes of the outcome measure, i.e., with partially applies-responses excluded and partially applies-responses coded as attained instead of (partially) not attained (for more information on coding, see Instrument Section). Both reiterations rendered the effect of externalizing MHP significant, *b* = 0.23, Wald χ^2^ (1, *N* = 380) = 4.07, *p* = 0.044 and *b* = 0.24, Wald χ^2^ (1, *N* = 433) = 4.51, *p* = 0.034, respectively. No changes were found for internalizing MHP. Externalizing and internalizing MHP and all control variables explained Nagelkerke's *R*^2^ = 0.26 and *R*^2^ = 0.24, respectively. This indicates a modest improvement in model fit over the baseline model with only the constant included; χ^2^ (9, *N* = 380) = 23.29, *p* = 0.006 and χ^2^ (9, *N* = 433) = 22.01, *p* = 0.009, respectively.

Rerunning model B with the interaction terms externalizing MHP by gender, internalizing MHP by gender, externalizing MHP by age, and internalizing MHP by age entered as predictors in an additional, final step of the hierarchical analysis confirmed our results on age and level of parental education and did not result in relevant changes (statistics not reported).

#### Dropout From Vocational or Academic Training

Externalizing and internalizing MHP and all control variables explained Nagelkerke's *R*^2^ = 0.07 in model C on predicting dropout from vocational or academic training. This indicates a poor improvement in model fit over the baseline model with only the constant included. Insignificant model fit of the final model [χ^2^ (9, *N* = 433) = 16.82, *p* = 0.052] restricts a reliable interpretation of findings on the insignificant association between externalizing and dropout [*b* = −0.08, Wald χ^2^ (1, *N* = 433) = 1.79, *p* = 0.181] and the significant association of internalizing MHP and dropout [*b* = 0.11, χ^2^ (1, *N* = 433) = 4.78, *p* = 0.029].

##### Sensitivity and Additional Analysis

Rerunning model C with different coding schemes, i.e., with partially applies-responses excluded and with partially applies-responses coded as no dropout vs. (partial) dropout (for more information on coding, see Instrument Section) did not improve model fit. Model fit of model C also remained insignificant after reiterating model C with the interaction terms externalizing MHP by gender, internalizing MHP by gender, externalizing MHP by age, and internalizing MHP by age entered as predictors in an additional, final step (statistics not reported).

We examined participants' subjective causal attributions about the relationship between MHP and reported dropouts. Results revealed a significant association between reporting *having MHP that led to falling short of one's potential in a course of education* and reporting dropout, χ^2^ (1, *N* = 433) = 28.54, *p* < 0.001, with a medium effect size of φ = 0.26. We also found a significant association between reporting *having or having had MHP that threatened successful completion of a course of education* and reporting dropout, χ^2^ (1, *N* = 433) = 23.05, *p* < 0.001, with a medium effect size of φ = 0.23. Reporting dropout and reporting that *a dropout was experienced due to MHP* were also significantly associated, as indicated by Fisher's exact test, *p* < 0.001, with a large effect size of φ = 0.58.

We reran all final Models A to C using only cases with complete data, confirming our results (statistics not reported).

## Discussion

To our knowledge, the present longitudinal study was the first in Germany to examine whether MHP in adolescence negatively affect educational attainment in early adulthood. Our findings partially confirmed our first hypothesis. More pronounced externalizing MHP among adolescents predicted an increased likelihood of attaining a lower level of education in early adulthood, while more pronounced internalizing MHP did not. Contrary to our second hypothesis, neither more pronounced externalizing MHP nor more pronounced internalizing MHP among adolescents predicted an increased likelihood of failing to attain the level of education that participants had expected to attain by early adulthood. If coding was varied as part of tests of robustness, externalizing MHP predicted an increased likelihood of failing to attain the expected level of education, while internalizing MHP did not. Concerning our third hypothesis on predicting dropout from vocational or academic training, insignificant model fit did not allow reliable interpretation of our findings. In our additional analysis, however, participants reported a subjective causal attribution between their MHP and their reported dropout. In addition, we observed that adolescents with higher-educated parents were less likely to attain a lower level of education themselves and less likely to fail in attaining their expected level of education. In sum, our findings indicate a detrimental impact of externalizing MHP in adolescence on the level of education in early adulthood. We specify recommendations for further investigation of the relationship between MHP in adolescence and educational attainment in early adulthood.

### Lower Level of Education

In line with previous findings, more pronounced externalizing MHP in adolescence predicted an increased likelihood of attaining a lower level of education in early adulthood. Symptoms of externalizing MHP, e.g., inattentiveness, impulsivity, or deviant behavior, can be perceived as disturbing and disruptive in the classroom (9, 32, 42). Externalizing MHP among adolescents are associated with engaging in risk-taking behaviors, such as substance use, readiness to use violence, or truancy which can in turn affect school performance and educational attainment (33). We suggest that future research replicates our findings on the association between externalizing MHP and attaining a lower level of education and considers possible mediating factors both within and outside the classroom.

In the present study, more pronounced internalizing MHP did not predict an increased likelihood of attaining a lower level of education. This is contrary to our hypothesis, but partly in line with existing literature pointing to stronger and more consistent effects of externalizing MHP on educational attainment compared to a more mixed picture for internalizing MHP (30, 34). For instance, while Evensen et al. (8) observed that the initial negative association between internalizing MHP and educational attainment disappeared when externalizing MHP were controlled for, Mikkonen et al. (34) found that internalizing MHP were negatively associated with educational attainment regardless of co-occurring externalizing MHP. In our study, we assessed MHP within a low-risk, epidemiological sample several years before school graduation. Considering the increasing trajectories of internalizing MHP across adolescence, symptoms of internalizing MHP, e.g., excessive worrying, or contact difficulties and withdrawal, may not have been pathological, i.e., pronounced enough, at baseline to exert a detrimental impact on educational attainment (42, 43). Haller et al. (33) reported that internalizing MHP were inversely associated with certain deviant or risk-taking behaviors. In addition, associations between MHP and educational attainment were less consistent for anxiety than for depression (40). We suggest that future research examines possible effects of depression and anxiety on educational attainment separately and in comparison. We recommend an examination of the distinct trajectories of externalizing and internalizing MHP across adolescence and to further assess associations with educational attainment both within low-risk epidemiological and within clinical samples. Considering the significant interaction effect of internalizing MHP and age in our additional analysis, future reaserach needs to disentangle the interplay of MHP, age and gender in more detail.

Our findings support that externalizing MHP impair educational attainment in the long-term. We emphasize that not only the detrimental effects of externalizing MHP, but also those of lower educational attainment, can accumulate over time and further disadvantage those affected by MHP. To help adolescents thrive in their transition to early adulthood, tailored measures to prevent the onset and persistence of MHP and to foster equal opportunities in education are needed.

### Failure to Attain the Expected Level of Education

Contrary to our second hypothesis, neither more pronounced externalizing MHP nor more pronounced internalizing MHP among adolescents predicted an increased likelihood of failing to attain the level of education that participants had expected to attain by early adulthood. Participants were asked about having or not having attained their expected level of education retrospectively. Early adults' recollections at the 6-year follow-up of their initial expectations at baseline assessment might have been biased in hindsight (77, 78). If coding was varied as part of tests of robustness, more pronounced externalizing MHP did predict an increased likelihood of failing to attain the expected level of education, whereas more pronounced internalizing MHP did not. The latter results are in line with our findings on the objective level of education. Contrasting the associations between externalizing and internalizing MHP in adolescence and the later attained *objective* level of education vs. the failure to attain the *subjectively* expected level of education can provide important insights to adolescents' self-awareness, self-image, and their capabilities to cope with MHP in the transition from adolescence to early adulthood (9, 28). The link between externalizing and internalizing MHP and the expected level of education deserves further investigation. We suggest that future research gathers data on the expected level of education already at baseline and utilizes a revised instrument to assess expectations regarding the future level of education.

### Dropout From Vocational or Academic Training

Concerning our third hypothesis on predicting dropout from vocational or academic training, insignificant model fit did not allow reliable interpretation of our findings on the association between externalizing and internalizing MHP and dropout. Findings from our additional analysis, however, showed a strong association between reporting a dropout and reporting that this dropout was caused by MHP. Potentially relevant data on other factors affecting dropout, such as family or classroom climate, levels of optimism, satisfaction with the course of education, or perceived stress were not gathered (47, 79, 80). Moreover, assessment of dropout from a course of education was worthy of improvement. While “course of education” [German original wording: “Ausbildung(-smaßnahme)”] is supposed to refer to vocational or academic training, it cannot be ruled out that participants' individual interpretations differed and included, e.g., dropout from clubs or seminars. We did not gather data on whether the decision to dropout was made voluntarily or not. We suggest that future research on the association between externalizing and internalizing MHP and dropout utilizes a more thorough operationalization of dropout from a “course of education” and gathers additional data on other relevant factors that might also affect dropout.

### Level of Parental Education

With an increasing level of parental education, adolescents in our study were less likely to attain a lower level of education themselves and less likely to fail in attaining their expected level of education. In line with Meyrose et al. (26), higher levels of parental education functioned as a resource in our sample. Our findings support that educational attainment presents the central channel for intergenerational reproduction of education and forms the main pathway for upward, but also downward social mobility (52–54).

### Strengths and Limitations

The present study contributes to the debate on the association between MHP and educational outcomes, especially in industrialized countries besides the United States (29–31). Longitudinal population-based data from Germany were used to investigate the joint impact of externalizing and internalizing MHP on educational attainment. A wide age range across the transition from adolescence to early adulthood, and from secondary school education to vocational or academic training was covered. As educational attainment during these transitions determines later opportunities in life, knowledge about major developmental transitions is fundamental for developing effective and tailored prevention measures. Finally, the present study assessed multiple measures of educational attainment: the objective level of education assessed by attained school-leaving certificates, attainment of the expected level of education, and dropout from vocational or academic training.

However, the following limitations should be noted: Assessment of failure to attain the expected level of education and of dropout from vocational or academic training are worthy of improvement. Phrasing of items should be more specific about the respective measure of educational attainment, e.g., by including a concise description, and only allow binary responses of either yes or no. Moreover, internal consistency of externalizing and internalizing MHP scores was only close to acceptable in the investigated sample; internal consistencies of the respective subscales were lower. We suggest that future research utilizes a more extensive instrument to assess externalizing and internalizing MHP in adolescence. The generalizability of our findings may be limited due to selective sampling of participants. We designed the sampling procedure to specifically cover the transition from adolescence to early adulthood, i.e., from secondary school education to vocational or academic training. We found small differences between our study sample and BELLA baseline participants with the same age range, but not meeting inclusion criteria. In line with findings from dropout analyses of the overall BELLA study (60), especially those participants with lower SES or with migration background were lost to follow-up. Externalizing MHP among our study sample were significantly less pronounced, implying that we did not include all affected participants. Finally, our study design did not allow for examining causal relationships.

### Future Directions

We suggest that future research uses concurrent evaluations of MHP and educational attainment with, e.g., annual or semi-annual assessments, so that specific educational and social transitions can be examined more closely and under consideration of relevant life events outside the school context. Considering comprehensive integrated schools for future research on the association between MHP and educational attainment seems promising. In comprehensive integrated schools students from all education tracks (*Hauptschule, Realschule*, and *Gymnasium*) are mostly taught together in mixed classes, regardless of ability level, similiar to the U.S. educational system. Finally, we suggest that future research explores underlying mechanisms that might affect the association between MHP and educational attainment, e.g., cognitive skills, school and class climate, and health and risk-taking behaviors.

#### Current Measures of Prevention and Recommendations for Future Practice

Effective prevention measures of MHP need to support adolescents during their transition from secondary education to vocational or academic training. Due to compulsory schooling in Germany, the school setting provides a central location for the implementation of prevention measures (6, 81, 82). The school setting overcomes a common dilemma, in that it gives all adolescents the same access to measures of prevention, including those who are systematically disadvantaged such as adolescents with lower-educated parents or with a migration background. Until today, measures of prevention in the German school context are heterogeneous and of diverse quality. No central network exists to catalog and evaluate such measures, although the German law on prevention and health promotion was revised and remitted in 2016 to incorporate prevention measures for mental health on a structural level, potentially also in the school setting (81). Paulus et al. developed a German version of the behavioral prevention program MindMatters (https://www.mindmatters-schule.de/). MindMatters is a scientifically supported and field-tested prevention program that utilizes classroom- and school-development modules to promote individual mental health among students. Nevertheless, dissemination of effective measures remains improvable (81, 82). Further, the rates of mental health care use among adolescents (9, 83) and rates of school psychologists, i.e., professionals to detect and accommodate for MHP in the school setting (6, 84), are low in Germany. To prevent the onset of MHP in adolescence on the level of individual behavioral prevention, we suggest increasing the rate of school psychologists and school social workers and familiarizing teachers with freely accessible prevention programs such as MindMatters. Furthermore, teachers should be trained to recognize warning signs of MHP and to refer affected children and adolescents for mental health services. On a structural level, we highlight the need for strategic public health investments to develop further effective prevention measures and to establish a central network that catalogs and disseminates these effective prevention measures.

## Conclusion

The present longitudinal study is the first to lend support to a detrimental impact of externalizing MHP in adolescence on educational attainment in early adulthood in Germany and initiates further research. Study results emphasize the importance of parental education and its downstream effects on offspring's education, the need to already apply prevention measures in adolescence, and to choose the school setting as a central location for implementation. Policy and institutions should realize this potential in order to prevent the onset and persistence of MHP, in order to help adolescents thrive in their transition to early adulthood, and, ultimately, to foster equal opportunities in education and beyond.

## Data Availability Statement

The original contributions presented in the study are included in the article/Supplementary Material, further inquiries can be directed to the corresponding author.

## Ethics Statement

The studies involving human participants were reviewed and approved by Ethics Committee of the University Hospital Charité Berlin and by the Federal Commissioner for Data Protection in Germany. Written informed consent to participate in this study was provided by the participants' legal guardian/next of kin. Additional written informed consent was provided by the participants aged 14 years or older.

## Author Contributions

CM, A-KM, AK, MM, and UR-S contributed conception and design of the study. CM and A-KM organized the database. CM wrote the first draft of the manuscript and performed the statistical analysis in close consultation with A-KM and MM. AK-M wrote sections of the manuscript. UR-S provided resources and conducted project administration and supervision. All authors contributed to manuscript revision, read, and approved the submitted version.

## Funding

This BELLA study has been financially supported by various grants: the German Science Foundation financed baseline, 1-year follow-up and 2-year follow-up assessments of the BELLA study. The German Federal Ministry of Health (BMG) funded the 6-year follow-up assessment.

## Conflict of Interest

The authors declare that the research was conducted in the absence of any commercial or financial relationships that could be construed as a potential conflict of interest.

## Publisher's Note

All claims expressed in this article are solely those of the authors and do not necessarily represent those of their affiliated organizations, or those of the publisher, the editors and the reviewers. Any product that may be evaluated in this article, or claim that may be made by its manufacturer, is not guaranteed or endorsed by the publisher.
