# Supplementary material for: Associations Between Mental Health Problems in Adolescence and Educational Attainment in Early Adulthood: Results of the German Longitudinal BELLA Study
Source: Front Pediatr. 2022 Feb 25;10:828085. doi: 10.3389/fped.2022.828085 (PMC8914221; doi:10.3389/fped.2022.828085)
Supplement: Supplementary file 1 [file Table_1.DOCX]

Supplementary Material

Supplementary Table 1

*Baseline characteristics (T0) of the study sample under analysis compared to BELLA baseline participants with the same age range, but without follow-up data*

|  | **Study sample**  (*n* = 433) | | | **Adolescents with the same age range, but without follow-up data**  (*n* = 1,288) | | | **Effect sizes of differences**  (independent samples t-test/  χ²-test) |
| --- | --- | --- | --- | --- | --- | --- | --- |
|  | ***n*** | **Valid %** | ***M* (*SD*)** | ***n*** | **Valid %** | ***M* (*SD*)** | **Cohen’s *d* / φ** |
| Gender | 433 |  |  | 1,288 |  |  | -0.06* |
| *Male* | 196 | 45.3 |  | 664 | 51.6 |  |  |
| *Female* | 237 | 54.7 |  | 624 | 48.4 |  |  |
| Age (in completed years with two decimals) | 433 |  | 14.88 (1.79) | 1,288 |  | 14,23 (2.05) | -0.33* |
| Migration background | 433 |  |  | 1,287 |  |  | 0.06* |
| *Yes* | 29 | 6.7 |  | 142 | 11.0 |  |  |
| *No* | 404 | 93.3 |  | 1,145 | 89.0 |  |  |
| Parental education (in years) | 427 |  | 13.17 (2.33) | 1,262 |  | 12.58 (2.20) | -0.26* |
| Household income (in 100€) | 427 |  | 12.41 (5.55) | 1,270 |  | 11.57 (5.83) | -0.15* |
| Parental status of employment | 432 |  |  | 1,275 |  |  | -0.05* |
| *At least one parent unemployed* | 49 | 11.3 |  | 200 | 15.7 |  |  |
| *None unemployed* | 383 | 88.7 |  | 1,075 | 84.3 |  |  |
| Externalizing MHP score | 432 |  | 5.09 (2.70) | 1,279 |  | 5.68 (2.90) | 0.21* |
| *Hyperactivity Scale* | 432 |  | 3.31 (1.94) | 1,279 |  | 3.71 (2.05) | 0.20* |
| *Conduct Problems Scale* | 432 |  | 1.78 (1.34) | 1,279 |  | 1.97 (1.38) | 0.14* |
| Internalizing MHP score | 432 |  | 4.27 (2.85) | 1,279 |  | 4.34 (2.78) | 0.03^n.s.^ |
| *Emotional Problems Scale* | 432 |  | 2.34 (1.98) | 1,279 |  | 2.39 (1.94) | 0.03^n.s.^ |
| *Peer Problems Scale* | 432 |  | 1.93 (1.55) | 1,279 |  | 1.95 (1.51) | 0.01^n.s.^ |
| *Note*. MHP = mental health problems. *M* = mean; *SD* = standard deviation; ^n.s.^ = non-significant difference, * *p* < .05 (*p* was Holm-Bonferroni corrected). | | | | | | | |
