# Supplementary material for: Associations Between Mental Health Problems in Adolescence and Educational Attainment in Early Adulthood: Results of the German Longitudinal BELLA Study
Source: Front Pediatr. 2022 Feb 25;10:828085. doi: 10.3389/fped.2022.828085 (PMC8914221; doi:10.3389/fped.2022.828085)
Supplement: Supplementary file 2 [file Table_2.docx]

Supplementary Material

| Supplementary Table 2. Bivariate associations among externalizing MHP, internalizing MHP, the control variables, and the outcome variables of educational attainments. | 12 | -.05 | .09 | .12* | -.01 | .12* | .05 | -.02 | -.05 | .06 | .04 | .10* | 1 | *Note*. *N* = 433. T0 = baseline assessment, T1 = 6-year follow-up, MHP = mental health problems. Missing values were given for *n* = 6 parental education, *n* = 6 household income, *n* = 1 parental status of employment, *n* = 1 externalizing MHP, and *n* = 1 internalizing MHP, and were replaced utilizing the EM-Algorithm. All coefficients of bivariate association are referred to as *r*, Pearson`s correlation coefficient, here, as Pearson`s correlation coefficient *r* (one continuous x one continuous variable) gives equal results as both the point-biserial correlation  *r*_pb_ (one continuous x one dichotomous variable) as well as φ (one dichotomous x one dichotomous variable) here, significant effects in bold.  **p* ≤ 05  ***p* ≤ 01. |
| --- | --- | --- | --- | --- | --- | --- | --- | --- | --- | --- | --- | --- | --- | --- |
|  | 11 | .14** | .09 | -.16** | .00 | -.08 | -.02 | -.17** | -.09 | -.01 | .30** | 1 |  |  |
|  | 10 | .19** | -.01 | -.15** | .06 | -.10* | -.11* | -.23** | -.16** | .10* | 1 |  |  |  |
|  | 9 | .06 | .04 | .03 | .00 | .04 | -.08 | -.10* | -.27** | 1 |  |  |  |  |
|  | 8 | -.08 | -.09 | .11* | .04 | .07 | .10* | .51** | 1 |  |  |  |  |  |
|  | 7 | -.12* | -.04 | .19** | .02 | .13** | -.06 | 1 |  |  |  |  |  |  |
|  | 6 | -.06 | -.09 | -.03 | .00 | .02 | 1 |  |  |  |  |  |  |  |
|  | 5 | -.05 | .02 | .68** | -.14** | 1 |  |  |  |  |  |  |  |  |
|  | 4 | .06 | -.25** | -.17** | 1 |  |  |  |  |  |  |  |  |  |
|  | 3 | -.12* | .10* | 1 |  |  |  |  |  |  |  |  |  |  |
|  | 2 | .21** | 1 |  |  |  |  |  |  |  |  |  |  |  |
|  | 1 | 1 |  |  |  |  |  |  |  |  |  |  |  |  |
|  |  | *r* | *r* | *r* | *r* | *r* | *r* | *r* | *r* | *r* | *r* | *r* | *r* |  |
|  |  | Externalizing MHP score (T0, centered) | Internalizing MHP score (T0, centered) | Age (in years, T0, centered) | Gender (T0, male) | Age*gender | Migration background (T0, yes) | Parental education (in years, T0, centered) | Household income (in 100€, T0, centered) | Parental status of employment (T0, at least one parent unemployed) | Level of education (T1, lower) | Level of education that was subjectively expected (T1, not attained) | Drop-out from a course of education (T1, yes) |  |
|  |  | 1 | 2 | 3 | 4 | 5 | 6 | 7 | 8 | 9 | 10 | 11 | 12 |  |
