# Supplementary material for: Associations Between Mental Health Problems in Adolescence and Educational Attainment in Early Adulthood: Results of the German Longitudinal BELLA Study
Source: Front Pediatr. 2022 Feb 25;10:828085. doi: 10.3389/fped.2022.828085 (PMC8914221; doi:10.3389/fped.2022.828085)
Supplement: Supplementary file 3 [file Table_3.DOCX]

Supplementary Material

Supplementary Table 3

*Additional analysis on predicting a lower level of education (A)*

|  | *B* | *SE* | *p* | *OR* | 95% CI of *OR* |
| --- | --- | --- | --- | --- | --- |
| Modified model A: lower level of education |  |  |  |  |  |
| Externalizing MHP (centered)*Gender | 0.10 | 0.16 | .543 | 1.10 | [0.81, 1.50] |
| Internalizing MHP (centered)*Gender | -0.21 | 0.19 | .275 | 0.81 | [0.56, 1.18] |
| Externalizing MHP (centered)*Age | 0.02 | 0.04 | .693 | 1.02 | [0.94, 1.10] |
| Internalizing MHP (centered)*Age | -0.13 | 0.06 | **.041** | **0.88** | [0.78, 1.00] |
| Externalizing MHP (centered) | 0.19 | 0.12 | .108 | 1.21 | [0.96, 1.52] |
| Internalizing MHP (centered) | -0.07 | 0.16 | .643 | 0.93 | [0.68, 1.27] |
| Age (in years; centered) | -0.34 | 0.19 | .070 | 0.71 | [0.49, 1.03] |
| Gender (male) | 0.17 | 0.57 | .772 | 1.18 | [0.38, 3.63] |
| Age*Gender | 0.02 | 0.26 | .944 | 1.02 | [0.61, 1.69] |
| Migration background (yes) | 1.53 | 0.71 | **.032** | **4.64** | [1.15, 18.77] |
| Parental education (in years; centered) | -0.70 | 0.20 | **.001** | **0.50** | [0.34, 0.74] |
| Household income (in 100€; centered) | -0.07 | 0.07 | .356 | 0.93 | [0.81, 1.08] |
| Parental status of employment (at least one parent unemployed) | 0.85 | 0.60 | .157 | 2.33 | [0.72, 7.50] |
| χ² (13, *N* = 433) = 63.23, *p* **< .001**, Nagelkerke’s *R²* = .34 | | | | | |

*Note.* MHP = mental health problems; significant p-values and corresponding Odds Ratios (*OR*) in bold.
